# A sex dependent association between the history of autoimmune disease and the development of pancreatic cancer: a case-control study of 32,640 patients

**DOI:** 10.3389/fonc.2025.1613787

**Published:** 2025-07-08

**Authors:** Sven H. Loosen, Frederik J. Hansen, Tom Luedde, Christoph Roderburg, Karel Kostev

**Affiliations:** ^1^ Department of Gastroenterology, Hepatology and Infectious Diseases, University Hospital Düsseldorf, Medical Faculty of Heinrich Heine University Düsseldorf, Düsseldorf, Germany; ^2^ Epidemiology, IQVIA, Frankfurt, Germany

**Keywords:** pancreatic cancer, PDAC, risk factor, autoimmune disease, inflammatory bowel disease, rheumatoid arthritis, systemic lupus erythematosus, multiple sclerosis

## Abstract

**Background:**

Pancreatic cancer is a highly lethal cancer with increasing incidence and poor prognosis due to late diagnosis. While several risk factors are known, evidence on a potential role of autoimmune diseases remains limited. Given the increasing prevalence of autoimmune diseases and their association with various malignancies, this study aims to investigate their potential association with pancreatic cancer.

**Methods:**

5,440 patients with a first diagnosis of pancreatic cancer and 27,200 propensity score matched individuals without cancer were identified from the Disease Analyzer database (IQVIA). The outcome of the study was the association between the diagnosis of pancreatic cancer and a patient´s history of autoimmune disease.

**Results:**

Inflammatory bowel disease (OR: 1.69; 95% CI: 1.34-2.12) and rheumatoid arthritis (OR: 1.20; 95% CI: 1.03-1.41) were significantly associated with increased odds of pancreatic cancer. The OR was 1.25 for systemic lupus erythematosus and 1.26 for multiple sclerosis without reaching a statistical significance. In sex-stratified analyses, inflammatory bowel disease was strongly associated with pancreatic cancer in women (OR: 2.14; 95% CI: 1.59-2.89) but not in men (OR: 1.24; 95% CI: 0.86-1.78). A positive association between rheumatoid arthritis and pancreatic cancer was also observed in women (OR: 1.26; 95% CI: 1.03-1.53) but not in men (OR: 1.09; 95% CI: 1.03-1.53-1.44). In addition, the ORs for SLE (1.82) and MS (1.45) were increased in women to a clinically relevant extent that did not reach the significance level of <0.05. A similar increase was not observed in male patients.

**Conclusion:**

Autoimmune disease may be associated with an increased risk of developing pancreatic cancer, particularly in women. This highlights the importance of addressing gender differences in medical practice, particularly in relation to disease screening and surveillance.

## Introduction

Pancreatic cancer is one of the deadliest cancers and a persistent global health challenge. It is the seventh leading cause of cancer-related death worldwide, with an estimated 495,773 new cases and 466,003 deaths recorded in 2020 alone (1, 2). The incidence of pancreatic cancer has been steadily increasing over the past few decades, reflecting changes in lifestyle and dietary habits (3). The prognosis for pancreatic cancer remains poor, with an overall 5-year survival rate of approximately 12% (4). Treatment strategies typically include surgery for localized pancreatic cancer, often in combination with neoadjuvant or adjuvant chemotherapy and, in some cases, radiotherapy (5–7). For metastatic pancreatic cancer, the focus shifts to systemic treatments, including chemotherapy, targeted therapies, and immunotherapy (8–11). One of the main reasons for the poor prognosis is the late diagnosis. It is therefore particularly important to identify risk factors so that high-risk patients can be screened more effectively. Established risk factors for the development of pancreatic cancer include smoking (12), both active and passive, and obesity (13). In addition, metabolic disorders such as type 2 diabetes mellitus (14) and chronic inflammation of the pancreas increase the risk (15). Excessive alcohol consumption also contributes to an increased risk (16). However, there is still a lack of clearly defined risk factors for which the implementation of targeted screening has a proven benefit.

Similar to the rising incidence of pancreatic cancer, the prevalence of autoimmune diseases has also increased (17). Autoimmune diseases, such as chronic inflammatory bowel disease, increase the risk not only of colorectal cancer but also of extraintestinal malignancies, including cholangiocarcinoma, skin cancer, hematological malignancies, genitourinary cancers, cervical cancer, and prostate cancer (18). The presence of rheumatoid arthritis may also increase the risk of cancer. A study of approximately 1.3 million women demonstrated that rheumatoid arthritis was significantly associated with an increased risk of lung, cervical, and oropharyngeal cancers, as well as hematological malignancies (19). The presence of systemic lupus erythematosus or multiple sclerosis is also associated with an increased risk of both hematologic malignancies and solid tumors, such as lung cancer (20, 21).

An link between these autoimmune diseases and the incidence of pancreatic cancer remains unclear, and the aim of this study is to investigate this relationship.

## Materials and methods

### Database

This study used data from the Disease Analyzer database (IQVIA). Details of this database have been published previously (22). In brief, the Disease Analyzer database contains data on demographic variables, diagnoses and prescriptions of outpatients from general practices in Germany. The database covers approximately 1300 general practices in Germany. The panel of practices included in the Disease Analyzer database has previously been shown to be representative of office-based practices in Germany (22). The database has been widely used for epidemiological studies in recent years including cancer studies (23, 24).

### Study population

**Figure 1:**
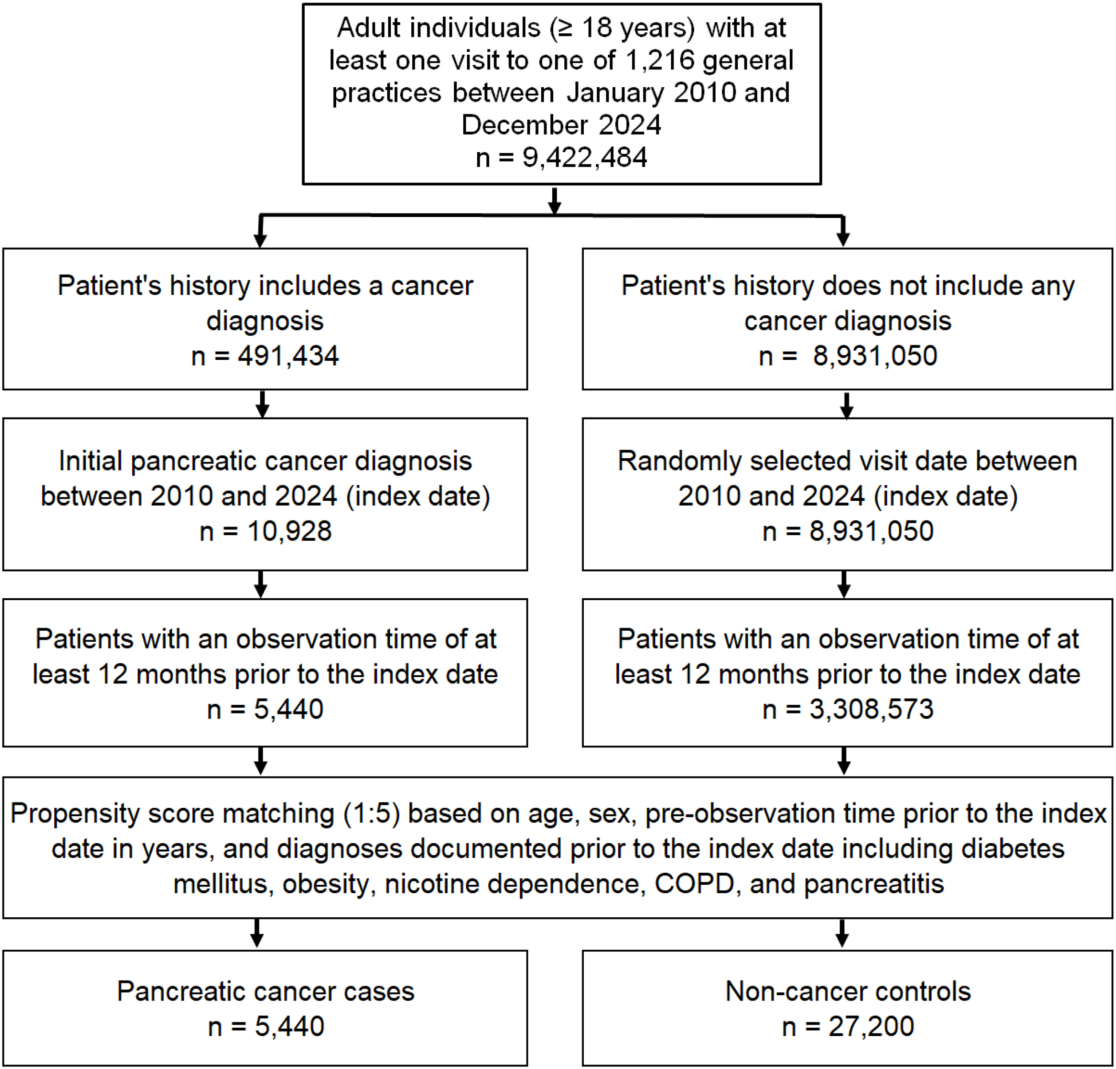
Selection of study patients.

The study population included all patients aged ≥18 years with a first diagnosis of pancreatic cancer (ICD-10 codes: C25) between January 2010 and December 2024 (index date) who had at least one year of follow-up before the index date. Controls were individuals without a history of cancer who were matched (5:1) by nearest neighbor propensity scores based on age, sex, pre-observation time prior to the index date in years, and documented diagnoses prior to the index date, including diabetes mellitus (ICD-10: E10-E14), obesity (ICD-10: E66), nicotine dependence (ICD-10: F17), chronic obstructive pulmonary disease (COPD) (ICD-10: J44), and history of pancreatitis (ICD-10: K85, K86). Diabetes, obesity, pancreatitis, and tobacco use are considered risk factors for pancreatic cancer (25). As information on tobacco use is not available, we used diagnoses of nicotine dependence and COPD which may proxy for smoking behavior. For individuals without cancer (controls), the index date was a randomly selected visit date between January 2010 and December 2024. The flow diagram of study participants is shown in **Figure 1**. For propensity score matching, a standardized mean difference (SMD) of less than 0.1 was allowed, indicating that adequate covariate balance has been achieved.

### Study outcome

The outcome of the study was the association between autoimmune diseases documented in the complete patient history before the index date and the diagnosis of pancreatic cancer. The diagnoses of interest were inflammatory bowel disease (IBD) (ICD-10: K50, K51), rheumatoid arthritis (ICD-10: M05, M06), psoriasis (ICD-10: L40), systemic lupus erythematosus (SLE) (ICD-10: M32), autoimmune thyroiditis (ICD-10: E06.3), and multiple sclerosis (ICD-10: G35).

### Statistical analyses

To examine whether the diagnosis of pancreatic cancer was associated with the patient history of autoimmune disease, we used multivariable conditional logistic regression models and estimated adjusted odds ratios (AORs) with 95% confidence intervals (95%CI). ORs were adjusted for age, sex, and documented diagnoses prior to the index date which were included in the PS matching. This model was also calculated separately for female and male patients. A p-value <0.05 was considered statistically significant. All analyses were performed using SAS version 9.4 (SAS Institute, Cary, US).

## Results

### Baseline characteristics

After 1:5 matching, 5,440 cases (patients with pancreatic cancer) and 27,200 controls (patients without cancer) were available for analyses. The mean age at the index date was 70.6 in patients with pancreatic cancer compared to 71.0 years in the control group. There was no significant differences between the two groups in terms of sex distribution. In both cohorts, 50% of the included patients were female. When examining the predefined co-diagnoses, there was no significant difference in the occurrence of diabetes mellitus between patients with pancreatic cancer and the control group. In contrast, patients with pancreatic cancer showed a slightly higher occurrence of overweight, COPD, nicotine dependence, and pancreatitis. On average, both cases and controls had 9.2 years of pre-observation time prior to index date. The prevalence of the most predefined co-diagnoses is shown in **Table 1**.

**Table 1:** Characteristics of study patients after 1:5 matching.

Variable	Pancreatic cancer (n=5,440)	Control patients (n=27,200)	SMD
Age (in years)			
Mean (SD)	70.6 (12.0)	71.0 (12.0)	-0.031
*≤* 60	983 (18.1)	4,757 (17.5)	
60-69	1,306 (24.0)	6,415 (23.6)	
70-79	1,751 (32.2)	8,733 (32.1)	
80 +	1,400 (25.7)	7.295 (26.8)	
Sex			
Female	2,725 (50.1)	13,660 (50.2)	-0.001
Male	2,715 (49.9)	13,540 (49.8)	
Observation time prior to the index date (years), mean (SD)	9.2 (5.8)	9.2 (5.8)	-0.009
Diagnoses documented prior to index date			
Diabetes mellitus	1,928 (35.4)	9,585 (35.2)	-0.002
Obesity	615 (11.3)	2,964 (10.9)	-0.004
COPD	853 (15.7)	4,124 (15.2)	-0.005
Nicotine dependence	407 (7.5)	1,816 (6.7)	-0.008
Pancreatis	672 (12.4)	3,146 (11.6)	-0.008

Data are absolute samples and percentages unless otherwise specified. SMD, standardized mean difference; SD, standard deviation.

### Association between the history of autoimmune diseases and pancreatic cancer diagnosis

**Table 2** shows the proportion of case and controls diagnosed with each of autoimmune disease as well as results of logistic regression analysis. In the total population, inflammatory bowel diseases (OR: 1.69; 95% CI: 1.34-2.12) and rheumatoid arthritis (OR: 1.20; 95% CI: 1.03-1.41) were associated with increased odds of developing pancreatic cancer. The OR was 1.25 for Systemic lupus erythematosus (SLE) and 1.26 for multiple sclerosis (MS), however, due to small samples of patients with these diseases, the p-value of <0.05 was not reached. The analysis of psoriasis and autoimmune thyroiditis showed no association with pancreatic cancer (**Table 2**).

**Table 2:** Association between autoimmune disorders and pancreatic cancer diagnosis in patients followed in general practices in Germany.

Variable	Patients with pancreatic cancer (%, n=5,440)	Control patients (%, n=27,200)	Adjusted OR (95% CI)	p-value
Inflammatory bowel diseases	1.84 (100)	1.08 (294)	1.67 (1.33-2.11)	<0.001
Rheumatoid arthritis	3.75 (204)	3.08 (838)	1.20 (1.03-1.41)	0.022
Psoriasis	3.51 (191)	3.13 (851)	1.11 (0.94-1.30)	0.211
Systemic lupus erythematosus	0.07 (4)	0.05 (14)	1.25 (0.41-3.84)	0.691
Autoimmune thyroiditis	2.57 (140)	2.52 (685)	1.00 (0.84-1.21)	0.965
Multiple sclerosis	0.44 (24)	0.34 (92)	1.26 (0.80-1.97)	0.322

OR, odds ratio; CI, confidence interval.

### A sex-depended association between autoimmune disease and pancreatic cancer

As autoimmune diseases are significantly more common in women, we subsequently performed a sex-stratified analysis (**Table 3**). Here, we found that inflammatory bowel disease was strongly associated with pancreatic cancer in women (AOR: 2.14; 95% CI: 1.59-2.89) but not in men (AOR: 1.24; 95% CI: 0.86-1.78). A positive association between rheumatoid arthritis and pancreatic cancer was also observed in women (AOR: 1.26; 95% CI: 1.03-1.53) but not in men (AOR: 1.09; 95% CI: 1.03-1.53-1.44). In addition, the ORs for SLE (1.82) and MS (1.45) were increased in women to a clinically relevant extent that did not reach the significance level of <0.05. A similar increase was not observed in male patients. The analysis of psoriasis and autoimmune thyroiditis revealed no significant differences between male and female patients.

**Table 3:** Association between sex, autoimmune disorders, and pancreatic cancer diagnosis among patients followed in general practices in Germany.

Variable	Patients with pancreatic cancer (%, n=5,440)	Control patients (%)	Adjusted OR (95% CI)	P value
Women				
Inflammatory bowel diseases	2.31(63)	1.07 (146)	2.14 (1.59-2.89)	<0.001
Rheumatoid arthritis	5.10 (139)	3.98 (544)	1.26 (1.03-1.53)	0.019
Psoriasis	3.08 (84)	2.94 (402)	1.03 (0.81-1.31)	0.825
Systemic lupus erythematosus	0.15 (4)	0.07 (10)	1.82 (0.55-6.01)	0.325
Autoimmune thyroiditis	4.22 (115)	4.09 (559)	1.02 (0.83-1.25)	0.874
Multiple sclerosis	0.73 (20)	0.49 (67)	1.45 (0.88-2.40)	0.149
Men				
Inflammatory bowel diseases	1.36 (37)	1.09 (148)	1.24 (0.86-1.78)	0.247
Rheumatoid arthritis	2.39 (65)	2.18 (295)	1.09 (0.83-1.44)	0.525
Psoriasis	3.94 (107)	3.32 (450)	1.19 (0.96-1.47)	0.121
Systemic lupus erythematosus	0.00 (0)	0.04 (5)	–	–
Autoimmune thyroiditis	0.92 (25)	0.94 (127)	0.97 (0.63-1.49)	0.870
Multiple sclerosis	0.15 (4)	0.19 (26)	0.75 (0.26-2.16)	0.598

OR, odds ratio; CI, confidence interval.

## Discussion

In summary, this study shows that the presence of IBD or RA was associated with increased odds of pancreatic cancer. Further analysis showed that this association was mainly observed in women and not in men.

Systemic inflammation plays a crucial role in the development and progression of pancreatic cancer. Chronic inflammatory states lead to a tumor-promoting microenvironment through the release of pro-inflammatory cytokines, chemokines, and growth factors. These mediators can induce DNA damage, promote malignant transformation, and facilitate tumor growth and immune evasion (26). Inflammatory pathways, such as NF-κB and STAT3, are often activated in pancreatic tissue under chronic inflammatory conditions, further promoting carcinogenesis. Persistent inflammation may also contribute to desmoplasia and fibrosis, both hallmarks of pancreatic ductal adenocarcinoma, creating a microenvironment conducive to tumor progression (27, 28). Unfortunately, the database does not contain markers of systemic inflammation, such as CRP or leukocyte counts. This is an important limitation of the present study.

In patients with inflammatory bowel disease systemic inflammation results primarily from chronic inflammation of the intestinal mucosa. In diseases such as Crohn’s disease and ulcerative colitis, the intestinal barrier is compromised, allowing microbial products such as lipopolysaccharides (LPS) to enter the bloodstream (29). This triggers an immune response beyond the gut, resulting in systemic inflammation. Key pro-inflammatory mediators involved include TNF-α, IL-6, IL-1β, and IFN-γ (30). These cytokines not only maintain local intestinal inflammation but also circulate systemically, activating immune cells and promoting a pro-tumorigenic environment in distant organs, including the pancreas. Chronic immune activation and cytokine signaling can lead to DNA damage, cellular stress, and changes in the tissue microenvironment that may contribute to carcinogenesis (31).

In rheumatoid arthritis, systemic inflammation results from an ongoing autoimmune response that primarily targets the synovial joints. This chronic immune activation leads to widespread inflammatory effects beyond the joints, driven by the sustained activation of immune cells such as macrophages, T cells, and B cells (32). As a result, pro-inflammatory cytokines, particularly TNF-α, IL-6, IL-1β, IL-23 and IL-17, are released into the systemic circulation (33). These mediators contribute to a chronic inflammatory state that can affect distant tissues and organs, promoting oxidative stress, endothelial dysfunction, and immune dysregulation, all of which can facilitate carcinogenesis in organs such as the pancreas. Another possible cause for the increased odd of pancreatic cancer could be the patients’ therapy. Most patients with RA receive disease-modifying antirheumatic drugs (DMARDs). It has already been shown that this immunosuppression can increase the risk of developing cancer (34); however, no study exists specifically addressing the development of pancreatic cancer. Unfortunately, our database does not contain information on whether and which patients received DMARDs. Therefore, follow-up studies are necessary to clarify this issue.

Systemic lupus erythematosus increases the risk of developing certain cancers, particularly non-Hodgkin lymphoma, due to chronic activation and dysregulation of the immune system (35). At the core of SLE is a breakdown in immune tolerance, driven primarily by overactive B cells. These cells, which normally produce antibodies to fight infection, become autoreactive in SLE, producing autoantibodies that mistakenly target the body’s own tissues (36). These cells show increased activation, resist normal cell death, and receive excessive survival signals such as BAFF. This persistent activation and expansion of B cells leads to systemic inflammation, persistent immune stimulation, and an increased risk of malignant transformation, particularly in lymphoid tissues (37). The role of B cells in the development of pancreatic cancer is not yet fully understood. However, there is evidence that B lymphocytes can secrete neoplastic growth factors and immunosuppressive cytokines, thereby promoting immune evasion and cancer progression (38). Furthermore, increased infiltration of B cells into the tumor has been associated with poorer patient prognosis (39). This supports the hypothesis that patients with SLE, which is characterized by overactive B cells, may also have an increased risk of developing pancreatic cancer. In our study, the number of patients with SLE was too small to detect a significant difference. Further studies are needed to clarify this possible association.

Women are disproportionately affected by autoimmune diseases such as inflammatory bowel disease and rheumatoid arthritis, a disparity that has been attributed to complex interactions between hormonal, genetic, and immunological factors. Estrogen and other sex hormones modulate immune responses by influencing cytokine production, T cell differentiation, and B cell activity (40). Studies have shown that estrogen enhances Th2-mediated immune responses and promotes the survival of autoreactive B cells, thereby increasing susceptibility to autoimmunity (41). In addition, many immune-related genes are located on the X chromosome, and women with two X chromosomes, are subject to incomplete X-chromosome inactivation, which may lead to overexpression of these genes (42, 43). Taken together, these findings suggest that the increased immune responsiveness in women, while beneficial in some contexts, predisposes them to a higher risk of developing autoimmune disease (43).

Our study results are consistent with the biological rationale that the presence of an autoimmune disease leads to increased systemic inflammation, thereby contributing to higher odds of developing pancreatic cancer. Interestingly, the overall incidence of pancreatic cancer is slightly higher in men than in women (44). This may be partly explained by the higher prevalence of risk factors such as smoking and alcohol consumption among men. However, autoimmune diseases also occur in men, although less frequently than in women. Interestingly, our sex-specific analysis showed that in men with a history of autoimmune disease, the odds of developing pancreatic cancer was not increased. The reason for this finding is unclear; it is possible that women exhibit a higher degree of systemic inflammation. This remains speculative and further underscores the need for sex-specific research.

Currently, there is no effective or widely recommended screening method for pancreatic cancer in the general population, primarily due to the disease’s typically late onset and lack of reliable early detection tools (7). However, screening may be considered for individuals at increased risk, including those with a strong family history of pancreatic cancer, known genetic syndromes (such as BRCA mutations or Lynch syndrome), or familial pancreatic cancer. Chronic pancreatitis is also recognized as a risk factor for pancreatic cancer, and patients with long-standing or hereditary pancreatitis may be considered for targeted surveillance. In high-risk individuals, imaging techniques such as endoscopic ultrasound (EUS) or magnetic resonance imaging (MRI) may be used, though their effectiveness in reducing mortality remains under investigation (45). However, further research is needed to determine whether women with a history of autoimmune disease should be screened for pancreatic cancer.

Our study has several limitations that are inherent to the database analysis and study design. First, we cannot exclude the possibility of misclassification of diagnoses or the absence of certain codes within the ICD‐10 coding system. Second, the database does not specify whether the pancreatic carcinoma patients have pancreatic ductal adenocarcinoma (PDAC) or neuroendocrine tumor (NET). This distinction would be important to know, as these entities differ significantly in their biological characteristics. In addition, the German Disease Analyzer Database does not include information on patients´ lifestyle, socioeconomic status or survival. It would be very interesting to know whether pancreatic cancer patients with a history of autoimmune disease have a different outcome compared to pancreatic cancer patients without a history of autoimmune disease. Furthermore, the case numbers for SLE and MS were too small to draw statistically significant results. In addition, not all major autoimmune diseases could be analyzed, because, for example, atopic dermatitis is rarely documented by general practitioners. As a result, it is difficult to make broad statements about autoimmune diseases in general.

However, the database provides a valuable perspective on general practitioner consultations in Germany, and the identification of inflammatory bowel disease and rheumatoid arthritis as factors associated with a pancreatic cancer in female patients may provide a basis for initiating further research on this topic.

In conclusion, we have shown that autoimmune diseases are associated with pancreatic cancer, particularly in women. This again highlights the need for greater attention to gender differences in medicine, especially in disease screening.

## Data Availability

The original contributions presented in the study are included in the article/supplementary material. Further inquiries can be directed to the corresponding author.
